# Acoustic Characteristics of Stridor in Multiple System Atrophy

**DOI:** 10.1371/journal.pone.0153935

**Published:** 2016-04-19

**Authors:** Dae Lim Koo, Jee Young Lee, Eun Yeon Joo, Seung Bong Hong, Hyunwoo Nam

**Affiliations:** 1 Department of Neurology, Seoul National University Boramae Hospital, Seoul, South Korea; 2 Department of Neurology, Samsung Medical Center, Sungkyunkwan University School of Medicine, Samsung Biomedical Research Institute, Seoul, South Korea; 3 Department of Health Sciences and Technology, SAIHST, Sungkyunkwan University, Seoul, South Korea; Florey Institute of Neuroscience and Mental Health, The University of Melbourne, AUSTRALIA

## Abstract

Nocturnal stridor is a breathing disorder prevalent in patients with multiple system atrophy (MSA). An improved understanding of this breathing disorder is essential since nocturnal stridor carries a poor prognosis (an increased risk of sudden death). In this study, we aimed to classify types of stridor by sound analysis and to reveal their clinical significance. Patients who met the criteria for probable MSA and had undergone polysomnography (PSG) were recruited. Patients were then assessed clinically with sleep questionnaires, including the Pittsburgh Sleep Quality Index, and the Hoehn and Yahr scale. Nocturnal stridor and snoring were analyzed with the Multi-Dimensional Voice Program. Nocturnal stridor was recorded in 22 patients and snoring in 18 patients using the PSG. Waveforms of stridors were classified into rhythmic or semirhythmic after analysis of the oscillogram. Formants and harmonics were observed in both types of stridor, but not in snoring. Of the 22 patients diagnosed with stridor during the present study, fifteen have subsequently died, with the time to death after the PSG study being 1.9 ± 1.4 years (range 0.8 to 5.0 years). The rhythmic waveform group presented higher scores on the Hoehn and Yahr scale and the survival outcome of this group was lower compared to the semirhythmic waveform group (p = 0.030, p = 0.014). In the Kaplan Meier’s survival curve, the outcome of patients with rhythmic waveform was significantly less favorable than the outcome of patients with semirhythmic waveform (log-rank test, p < 0.001). Stridor in MSA can be classified into rhythmic and semirhythmic types and the rhythmic component signifies a poorer outcome.

## Introduction

Multiple system atrophy (MSA) is a neurodegenerative disorder characterized by the combined symptoms of parkinsonism, cerebellar dysfunction, and autonomic failure [1]. Up to 70% of MSA patients complain of sleep disorders including sleep fragmentation, insomnia, REM sleep behavior disorder (RBD), obstructive sleep apnea (OSA), and nocturnal stridor [2]. Among these sleep disorders, nocturnal stridor has special significance since it is associated with a shortened survival period. Stridor is an easily recognizable harsh and strained high-pitched sound, which can be inspiratory, expiratory, or biphasic; usually, the sound is inspiratory in nature [3]. Selective paralysis of the vocal cord abductor is known to contribute to the development of nocturnal stridor [4]. Several laryngoscopic studies have revealed that glottal narrowing during inspiration can cause laryngeal stridor [5, 6]. Several previous studies have reported shortened survival after the initiation of stridor [4, 7]. However, factors that may be predictive of longevity in patients with MSA are still uncertain.

Here, we have focused on the sound characteristics of stridor, since the narrowed airway may produce different sounds depending on the degree of muscle contraction of the vocal cord. The aim of this study was to differentiate nocturnal stridor from snoring and to classify stridors into different types through sound analyses. Moreover, we hypothesized that stridor classification using this method might be predictive of MSA patient outcome.

## Materials and Methods

### Subjects

Twenty-two patients with nocturnal stridor were recruited from the Seoul National University Boramae Hospital and the Samsung Medical Center, Seoul. Each patient completed a detailed clinical interview and a sleep questionnaire, and underwent overnight polysomnography (PSG). Subjective daytime sleepiness was measured with the Epworth Sleepiness Scale (ESS) and Stanford Sleepiness Scale (SSS). The Pittsburgh sleep quality index (PSQI) was used to measure the quality and disturbances of sleep during the last one month [8]. Clinical and PSG data were reviewed retrospectively and the rhythmicity of stridor was evaluated as a potential prognostic factor for survival outcome. Clinical diagnostic criteria for probable MSA, including autonomic failure plus parkinsonism or cerebellar ataxia, were fulfilled in all subjects [9]. Approval for this study was obtained from the Institutional Review Board at the Boramae Hospital of Seoul National University and Samsung Medical Center. We obtained written informed consent for participation from each patient or his/her legal representative.

### Overnight polysomnography

All patients underwent standard overnight PSG. Sleep studies were recorded using one of two PSG systems (Grass-Telefactor, USA and Embla, USA). The procedure was performed using a 6-channel electroencephalogram (EEG; F3/A2, F4/A1, C3/A2, C4/A1, O1/A2, and O2/A1), a 4-channel electrooculogram (EOG), electromyogram (EMG; submental and anterior tibialis muscles), and an electrocardiogram. Thermistor, nasal air pressure channel, oximeter, piezoelectric bands, and body position sensor were also applied. Behaviors and sounds from the subjects throughout the night were recorded using an infrared video camera and a neck microphone. Apneas and hypopneas were also scored, and the apnea-hypopnea index (AHI) was calculated. Obstructive apnea was defined as a reduction in airflow ≥ 90% lasting ≥ 10 s with an evidence of persistent respiratory effort. Hypopnea was defined as a reduction of airflow by ≥ 30% lasting ≥ 10 s, accompanied by a ≥ 4% oxygen desaturation [10].

### Analysis of stridor and snoring

Computerized Speech Lab software with a 4300 external module (Kay Elemetrics Corporation) was employed to perform objective voice evaluation. Voices were recorded with a microphone positioned approximately 15 cm from the mouth and slightly below the chin. All sound samples were digitized at 44,100 Hz with a 16-bit quantization level in stereo. A 30-ms audio sample of nocturnal sound and snoring were analyzed using the Multi-Dimensional Voice Program. Oscillograms and spectrograms of each nocturnal sound were analyzed, oscillogram analysis included sound intensity and waveform data and spectrogram analysis comprised sound frequencies from 0 to 4,500 Hz. We subsequently estimated parameters such as fundamental frequency, perturbation of frequency (jitter), perturbation of amplitude (shimmer), and the presence of harmonics and formants. Analysis of the waveform was performed according to the criteria defined by Yanagihara [11]. According to those criteria, types I and II correspond to periodic or (almost) regular sounds generated by the vocal cords, whereas type III corresponds to irregular or chaotic sounds. The presence of formants and harmonics was confirmed by spectrogram (where formants correspond to a resonance in the vocal tract and harmonics are represented by a periodic signal that is an integer multiple of the fundamental frequency).

### Statistical Analysis

We used t-tests for continuous variables and Pearson’s χ2 and Fisher’s exact tests for categorical variables. All the continuous quantitative variables were noted as the mean and standard deviation (SD). A Kaplan-Meier survival curve was used to estimate the time-to-death outcomes according to the stridor pattern of sound analysis. Statistical significance was tested, using the log-rank test and comparison of 95% confidence intervals (CIs). Statistical analyses were performed with SPSS statistical software version 21 (SPSS Inc.). Two-sided p-values less than 0.05 were considered statistically significant.

## Results

### Clinical characteristics

The mean age of the patients at MSA symptom onset was 56.1 ± 6.7 years and at stridor onset was 59.0 ± 7.7 years, with a mean interval between the two of 2.9 ± 2.1 years. The age at PSG was 61.5 ± 6.8 years (range; 50 to 72 years). At the time of PSG, the duration of MSA symptom was 5.5 ± 2.0 years (range 2.8 to 9.5) and the duration of stridor was 2.6 ± 1.5 years (range 0.7 to 6.1). Parkinsonian features were predominant in 13 (59.1%) patients (MSA-P subtype) and cerebellar ataxia in 9 (40.9%) patients (MSA-C subtype). At the time of PSG, orthostatic hypotension was present in 14 (64%) patients and urinary dysfunction in 15 (68%). The Hoehn and Yahr stage was 1.9 ± 0.5 (range 1.0 to 3.0) at the time of the PSG study. The clinical characteristics of our patients are provided (S1 Table).

### Results of the sound analysis and PSG

**Fig 1:**
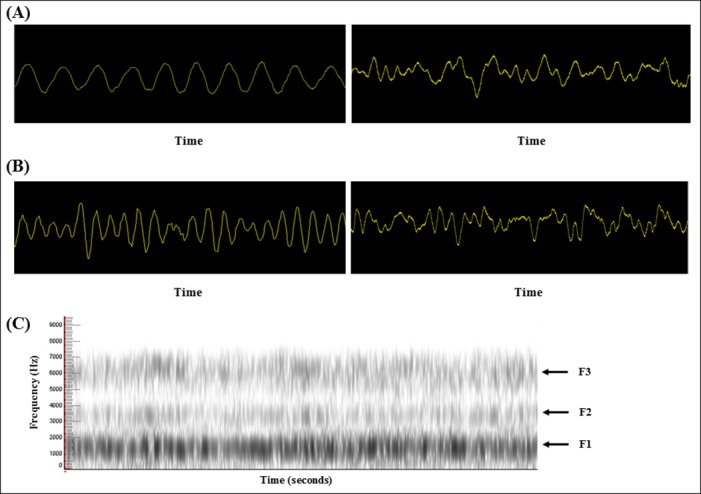
Oscillogram and spectrogram of nocturnal stridors. Two different types of waveforms in nocturnal stridor are described and the snoring signal of each patient was analyzed. Each sample has a duration of 40 ms. (A) Stridor in patient No. 3 presents a rhythmic sinusoidal waveform with a frequency of 250 Hz (left). The signal of snoring in patient No. 3 was irregular and non-periodic (right). (B) Oscillogram of patient No. 8 shows a semirhythmic signal (left). Analysis of snoring in patient No. 8 shows a chaotic signal (right). (C) Spectrogram of patient No. 2 shows three formants. The first (F1), second (F2), and third (F3) formants are indicated by black arrows.

Nocturnal stridors were evaluated by analyzing the oscillogram and spectrogram. The mean fundamental frequency was 193.8 ± 51.9 Hz (male, 168.6 ± 42.9; female, 229.1 ± 42.2). The jitter was 9.4 ± 2.5% and the shimmer was 14.6 ± 4.3%. All stridors exhibited the formation of formants and harmonics, which were demonstrated in the spectrogram. According to Yanagihara’s classification, nine patients showed nocturnal stridor with rhythmic waveform (type I), and 13 displayed the semirhythmic waveform (type II). Snoring in 18 patients presented irregular shaped sound (type III) with no formants and harmonics (Fig 1). Individual sound analysis and each PSG data are included (S2 Table).

We divided our patients into two groups according to the rhythmicity of stridor as determined via sound analysis. Patients presenting a rhythmic waveform showed worse survival overall and a shorter follow-up period after PSG in comparison with patients presenting a semirhythmic waveform (p = 0.014). The Hoehn and Yahr stage was significantly higher in the rhythmic waveform group compared to the semirhythmic waveform group (p = 0.030). However, the duration of stridor and MSA symptoms before PSG was not significantly different between the rhythmic and semirhythmic waveform groups (2.8 ± 1.4 years, 2.5 ± 1.6 years). Table 1 summarizes the clinical features of the two nocturnal stridor groups (rhythmic and semirhythmic).

**Table 1:** Clinical characteristics in patients with nocturnal stridor.

Variables	Patients (N = 22)	Rhythmic waveform (N = 9)	Semirhythmic waveform (N = 13)	p value
**Clinical factors**				
Men, No. (%)	12 (54.5)	5 (55.6)	7 (53.8)	0.938
Age of MSA symptom onset, years, mean (SD)	56.1 (6.7)	53.8 (7.5)	56.4 (5.6)	0.664
Age of stridor onset, years, mean (SD)	59.0 (7.7)	56.2 (8.7)	59.5 (6.7)	0.764
Interval between MSA symptom onset and stridor onset, years, mean (SD)	2.9 (2.1)	2.4 (2.2)	3.1 (2.2)	0.385
Age at PSG study, years, mean (SD)	61.5 (6.8)	59.0 (7.8)	62.0 (5.8)	0.664
Time from the stridor onset to PSG study, years, mean (SD)	2.6 (1.5)	2.8 (1.4)	2.5 (1.6)	0.504
Time to death or last visit after PSG, years, mean (SD)	1.9 (1.4)	1.3 (1.3)	2.3 (1.4)	**0.014**
Total duration of MSA, year, mean (SD)	7.4 (1.9)	6.5 (1.5)	7.9 (2.0)	0.061
Total duration of stridor, years, mean (SD)	4.5 (1.8)	4.1 (1.5)	4.8 (2.0)	0.350
BMI, kg/m^2^, mean (SD)	24.8 (2.6)	24.9 (3.5)	25.1 (2.0)	0.367
MSA subtype, MSA-P: MSA-C, No.	13: 9	4: 5	9: 4	0.256
Hoehn and Yahr scale, mean (SD)	1.9 (0.5)	2.2 (0.5)	1.6 (0.4)	**0.030**
ESS score, mean (SD)	8.2 (5.0)	9.4 (6.1)	7.8 (4.1)	0.947
SSS score, mean (SD)	3.0 (0.9)	3.5 (1.2)	2.6 (0.8)	0.192
Global PSQI score, mean (SD)	6.9 (2.4)	7.0 (2.6)	6.7 (2.6)	0.735
CPAP therapy, No. (%)	8 (36.4)	3 (33.3)	5 (38.5)	0.845

BMI, body mass index; MSA, multiple system atrophy; MSA-P, Parkinsonian type of MSA; MSA-C, cerebellar type of MSA; ESS, Epworth sleepiness scale; SSS, Stanford sleepiness scale; PSQI, Pittsburgh sleepiness quality index; CPAP, continuous positive airway pressure

Twenty-two patients had a definite sleep-disordered breathing diagnosis (mean AHI, 21.6/hr, ranged 0.0 to 102.8). Ten patients had periodic limb movements during sleep (PLMS; total PLM index, 86.5/h), and 14 patients revealed REM sleep behavior disorder. Nocturnal stridors were noticed during PSG in all patients. Snoring was recorded in 18 participants. There was no significant difference between the rhythmic and semirhythmic waveform groups with regard to the acoustic and PSG variables (Table 2).

**Table 2:** Acoustic and PSG characteristics of nocturnal stridor.

Variables	Patients (N = 22)	Rhythmic waveform (N = 9)	Semirhythmic waveform (N = 13)	p value
**Acoustic factors**				
Sound volume, dB, mean (SD)	61.6 (6.8)	64.3 (6.6)	60.8 (6.6)	0.423
Fundamental frequency, Hz, mean (SD)	193.8 (51.9)	195.2 (62.2)	198.5 (48.8)	0.815
PPQ, %, mean (SD)	8.2 (3.1)	8.4 (3.6)	7.9 (3.1)	0.973
APQ, %, mean (SD)	19.4 (5.0)	19.6 (5.2)	19.0 (5.4)	0.894
Jitter, %, mean (SD)	9.4 (2.5)	10.0 (2.1)	8.9 (2.8)	0.570
Shimmer, %, mean (SD)	14.7 (4.2)	16.9 (5.3)	13.9 (2.5)	0.204
NHR, mean (SD)	0.4 (0.2)	0.4 (0.3)	0.4 (0.2)	0.324
**Polysomnographic factors**				
Total sleep time, min, mean (SD)	298.4 (65.6)	321.3 (54.2)	269.5 (72.3)	0.133
N1 sleep, %, mean (SD)	17.1 (14.9)	20.4 (21.3)	16.3 (9.3)	0.526
N2 sleep, %, mean (SD)	52.6 (14.7)	54.2 (16.5)	54.3 (13.9)	0.920
N3 sleep, %, mean (SD)	13.3 (11.4)	7.8 (8.9)	14.9 (12.5)	0.169
REM sleep, %, mean (SD)	17.1 (10.2)	17.6 (8.7)	14.4 (9.5)	0.217
Sleep latency, min, mean (SD)	19.4 (16.6)	19.8 (16.8)	22.4 (21.8)	0.867
REM sleep latency, min, mean (SD)	136.7 (79.2)	119.9 (55.2)	148.0 (90.7)	0.243
Sleep efficiency, %, mean (SD)	70.3 (14.0)	73.4 (11.4)	65.2 (17.0)	0.151
Arousal index, events/h, mean (SD)	13.6 (12.8)	16.5 (13.9)	13.2 (11.9)	0.738
PLMS index, events/h, mean (SD)	35.0 (54.6)	75.1 (65.3)	20.3 (42.7)	0.080
AHI, events/h, mean (SD)	21.6 (24.5)	25.8 (34.8)	20.3 (16.2)	0.616
Longest sleep apnea, sec, mean (SD)	20.8 (21.7)	30.6 (30.0)	14.6 (12.5)	0.360
Lowest saturation of oxygen, %, mean (SD)	86.4 (3.4)	86.9 (3.4)	86.6 (2.8)	0.943

PPQ, pitch perturbation quotient; APQ, amplitude perturbation quotient; NHR, noise-to-harmonic ratio; PLMS, periodic limb movements during sleep; AHI, apnea-hypopnea index

### Survival outcome in patients with stridor

**Fig 2:**
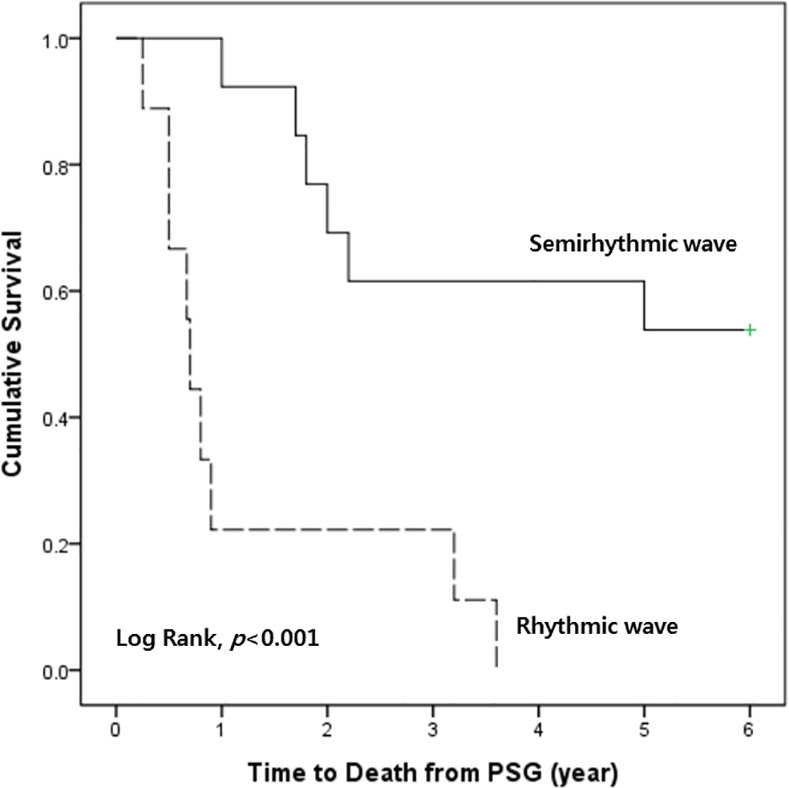
Kaplan-Meier analysis of waveform-specific survival rate in patients with MSA stridor. Patients with rhythmic waveform (n = 9) have a significantly worse prognosis than those with semirhythmic waveform (n = 13) (Log rank test, p<0.001)

Of the 22 patients with nocturnal stridor included in this study, 15 have subsequently died, with the time to death from the PSG being 1.7 ± 1.4 years (range 0.8 to 5.0 years). The seven survivors were followed up for 2.4 ± 1.4 years after the PSG. We applied correlation analysis between RBD and each type of stridor. There was no significant relationship between RBD and stridor subtypes (P = 0.131). Kaplan Meier’s survival analysis was applied to compare the survival after PSG between rhythmic and semirhythmic waveform groups. Patients with rhythmic waveform demonstrated a significantly more unfavorable outcome than those with semirhythmic waveform (log-rank test, p < 0.001) (Fig 2).

## Discussion

In the present study, we have demonstrated that: (1) nocturnal stridors of MSA could be classified into two types on the basis of acoustic rhythmicity; and (2) rhythmic waveform may be a useful predictor for short-term survival as demonstrated in Kaplan Meier’s curves. To the best of the authors’ knowledge, this is the first report that has described the acoustic characteristics of nocturnal stridor in patients with MSA and demonstrated that waveform was predictive of survival outcome.

MSA is a progressive fatal neurodegenerative disorder. Indicators predicting survival in patients with MSA have not been successfully established. Yamaguchi et al. reported that the median survival period in patients with MSA stridor was 8.0 years and the stridor developed 2.7 ± 2.3 years after the onset of MSA [12]. The clinical characteristics of patients in our study were similar to those of the Yamaguchi study, with a median survival period of 7.3 ± 2.0 years and a mean time interval of 2.9 ± 2.1 years between MSA and stridor onsets. Nocturnal stridor has been considered to be a predictor of unfavorable prognosis in MSA [4, 7]. Silber et al. reported that six patients died a mean of 2.4 years after evaluation at the sleep center [4]. Our patients with stridor showed a short mean survival period of 1.7 ± 1.4 years after the PSG. Sex and phenotype of MSA (MSA-P vs. MSA-C) did not significantly different between rhythmic and semirhythmic waveform groups. The observation that there was no significant relationship between sex or MSA subtype and survival outcome in our study was consistent with some previous reports, [13, 14] but inconsistent with others [15–18]. A recent study reported that sex, phenotype, and early development of gait instability were not indicators of shorter survival [14]. Instead, early development of severe generalized autonomic failure was indicative of shorter survival in patients with MSA. However, the authors did not consider the effect of stridor on survival since stridor was only proven in a small number of patients. The duration of stridor and MSA symptoms at the time of PSG was not significantly different between rhythmic and semirhythmic waveform groups in our study. Nevertheless, the rhythmic waveform group displayed a higher Hoehn and Yahr score and a worse survival outcome compared to the semirhythmic waveform group, suggesting these waveform groups comprise two new subtypes of MSA. Continuous positive airway pressure (CPAP) treatment is an effective treatment for the elimination of sleep disordered breathing in patients with MSA [19]. The effect of this treatment on the survival or morbidity of MSA is not known. Previously, several studies have reported that severity of motor impairment and vulnerability to pulmonary infection may be limiting factors for the maintenance of CPAP treatment [20, 21]. However, in the present study, any ambiguity in the effect of CPAP treatment needed not be considered, since the proportion of CPAP therapy between rhythmic and semirhythmic waveform groups was not significantly different.

Patients with MSA frequently manifest a variety of sleep-related disorders including OSA, RBD, periodic limb movements in sleep (PLMS), and stridor [3, 22]. Our MSA patients with stridor also displayed OSA (77%), RBD (63%), and PLMS (45%). Among these sleep-related disorders, nocturnal stridor, classified as a sleep-related laryngospasm, is the most life-threatening condition [23]. A recent study has asserted that the severity of impairment of vocal fold motion negatively affected overall survival in patients with MSA, and that the presence of stridor without vocal cord paresis was not associated with an adverse prognosis [7]. In that study, stridors and other vocal fold problems were diagnosed only by an otolaryngologic examination, not by any physiological measures during sleep. In the current study, the sound source of stridor was extracted from naturally sleeping individuals, hence our method should be considered more physiological in comparison to the previous study.

The first type of nocturnal stridor that could be identified was characterized by a rhythmic waveform with regular frequency and amplitude, while the second type was characterized by a semirhythmic waveform with less formulaic frequency and amplitude. Formants are resonances of the vocal tract and the presence of harmonics is indicative of a vocal origin [24]. In our study, both types of stridor were comprised of formants and harmonics, thus suggesting that the nocturnal stridor is generated in the region of the vocal cord. In contrast, sound analysis of snoring in the previous studies revealed no formation of harmonics [24, 25]. The chaotic signal of snoring observed in our patients is in accordance with these studies. Previous studies which have used video laryngoscopes reported that the vocal cord might be the origin of MSA stridor [5, 7, 26]. Two additional acoustic parameters involving the vocal cord were also evaluated. Whereas jitter is a measure of the percentage of irregularity in the frequency of vocal sound, shimmer is a measure of the percentage of irregularity in the amplitude of vocal sound. Jitter primarily reflects the stability of vocal fold vibration and shimmer indicates the regularity of glottis dynamics (particularly glottic closure). Every stridor showed higher jitter and shimmer values, which is indicative of pathological conditions in vocal fold vibration and in the mucosal wave. Jitter and shimmer, like other acoustic parameters (apart from the waveform), are not significantly associated with survival outcome.

A previous study with laryngoscopy and video monitoring suggested that the presence of tremulous arytenoid movement was associated with the severity of glottis stenosis, and that this is clearly correlated with disease duration in MSA patients [27]. The occurrence of rhythmic waveform could be another marker of severe glottis stenosis. Although the pathogenic mechanisms underlying MSA stridor are still controversial, laryngeal abductor weakness or laryngeal adductor dystonia are considered a cause of stridor in MSA patients [28]. Neurogenic atrophy of the posterior cricoarytenoid muscle suggested the laryngeal abductor weakness, whereas dysfunction in the inhibitory brainstem autonomic pathways may cause dystonia in the laryngeal adductor muscles [27–29]. In this study, patients who displayed rhythmic signals had a worse outcome than those exhibiting less rhythmic waveform. We suggest that rhythmic waveform is indicative of a more enhanced laryngeal muscle tone or more severe glottic stenosis (as compared to semirhythmic waveform). The small sample size and the cross-sectional design may be considered limitations of this study. However, it is evident that sound analysis of stridor for rhythmicity is a less invasive, more physiological method for the analysis of stridor (compared to the laryngoscopic procedure).

In conclusion, stridor in MSA can be decomposed into rhythmic and semirhythmic waveforms, and the rhythmic component suggests a poorer outcome. Further longitudinal studies need to be performed to confirm whether this constitutes a specific type of stridor, or whether this is an innate characteristic of patients, of the disease, or of disease progression.

## Supporting Information

S1 Table Clinical demographics of nocturnal stridor. (DOCX)

S2 Table Polysomnographic and acoustic characteristics of nocturnal stridor. (DOCX)
