# Supplementary material for: Acoustic Characteristics of Stridor in Multiple System Atrophy
Source: PLoS One. 2016 Apr 19;11(4):e0153935. doi: 10.1371/journal.pone.0153935 (PMC4836672; doi:10.1371/journal.pone.0153935)
Supplement: S1 Table — (DOCX) [file pone.0153935.s001.docx]

**S1 Table. Clinical demographics of nocturnal stridor.**

| **Patient** | **Sex** | **Age (yrs)** | **BMI (kg/m2)** | **MSA subtype** | **HY stage** | **Time P (yrs)** | **Time S (yrs)** | **Time S before PSG (yrs)** | **Follow-up after PSG (yrs)** | **Status of last visit** | **Survival outcome^a^** |
| --- | --- | --- | --- | --- | --- | --- | --- | --- | --- | --- | --- |
| **1** | M | 70 | 21.5 | MSA-P | 2 | 4.5 | 2.5 | 2.0 | 0.5 | Dead | Long |
| **2** | M | 58 | 28.1 | MSA-C | 1 | 7.7 | 3.5 | 1.0 | 2.5 | Alive |  |
| **3** | F | 72 | 27.6 | MSA-P | 2 | 9.2 | 2.9 | 0.7 | 2.2 | Dead | Short |
| **4** | M | 68 | 23.5 | MSA-P | 2 | 7.4 | 5.9 | 1.9 | 4.0 | Alive |  |
| **5** | M | 63 | 26 | MSA-P | 1.5 | 5.9 | 3.7 | 2.0 | 1.7 | Dead | Short |
| **6** | M | 66 | 23.2 | MSA-C | 2 | 8.6 | 4.3 | 2.3 | 2.0 | Dead | Short |
| **7** | F | 62 | 26.2 | MSA-P | 1 | 10.5 | 3.1 | 2.1 | 1.0 | Dead | Short |
| **8** | F | 61 | 24.9 | MSA-C | 2 | 8.0 | 3.9 | 3.0 | 0.9 | Dead | Long |
| **9** | M | 70 | 20.5 | MSA-C | 1.5 | 3.5 | 2.9 | 2.2 | 0.7 | Dead | Long |
| **10** | F | 68 | 24.4 | MSA-P | 1 | 10.0 | 4.9 | 3.1 | 1.8 | Dead | Short |
| **11** | F | 62 | 23.9 | MSA-P | 2 | 11.1 | 9.1 | 5.3 | 3.8 | Alive |  |
| **12** | F | 67 | 25.7 | MSA-P | 2 | 5.6 | 2.1 | 1.3 | 0.8 | Alive |  |
| **13** | M | 53 | 26.0 | MSA-C | 2 | 5.9 | 5.0 | 4.2 | 0.8 | Dead | Long |
| **14** | F | 55 | 23.4 | MSA-C | 2 | 6.8 | 4.7 | 1.1 | 3.6 | Dead | Short |
| **15** | F | 52 | 21.5 | MSA-C | 1.5 | 8.0 | 7.3 | 6.1 | 1.2 | Alive |  |
| **16** | F | 64 | 32.0 | MSA-P | 2 | 7.3 | 2.2 | 1.5 | 0.7 | Dead | Long |
| **17** | M | 69 | 26.0 | MSA-P | 2 | 8.0 | 2.6 | 2.1 | 0.5 | Dead | Long |
| **18** | F | 51 | 24.8 | MSA-C | 3 | 6.4 | 5.5 | 5.2 | 0.3 | Dead | Long |
| **19** | M | 54 | 26.6 | MSA-P | 1.5 | 6.6 | 6.0 | 2.5 | 3.5 | Alive |  |
| **20** | M | 50 | 22.0 | MSA-P | 3 | 6.4 | 6.4 | 3.2 | 3.2 | Dead | Short |
| **21** | M | 60 | 23.2 | MSA-C | 2 | 8.2 | 6.0 | 1.0 | 5.0 | Dead | Short |
| **22** | M | 56 | 26.8 | MSA-P | 1.5 | 4.3 | 3.8 | 3.0 | 0.8 | Alive |  |

BMI, body mass index; MSA, multiple system atrophy; MSA-P, Parkinsonian type of MSA; MSA-C, cerebellar type of MSA; HY stage, Hoehn and Yahr stage at the time of the PSG study; Time P, total duration of parkinsonian symptom onset to death or last visit; Time S, total duration of stridor onset to death or last visit; Time S before PSG, Duration from stridor onset to PSG confirmation

^a^ Long, the patient survived more than one year after PSG study; Short, the patients had died within one year after PSG study
