# Supplementary material for: Acoustic Characteristics of Stridor in Multiple System Atrophy
Source: PLoS One. 2016 Apr 19;11(4):e0153935. doi: 10.1371/journal.pone.0153935 (PMC4836672; doi:10.1371/journal.pone.0153935)
Supplement: S2 Table — (DOCX) [file pone.0153935.s002.docx]

**S2 Table. Polysomnographic and acoustic characteristics of nocturnal stridor.**

| **Patient** | **Polysomnographic results** | | | | | | | | | | | | | **Sound analysis of stridor** | | | | | | | | | |
| --- | --- | --- | --- | --- | --- | --- | --- | --- | --- | --- | --- | --- | --- | --- | --- | --- | --- | --- | --- | --- | --- | --- | --- |
|  | **TST (min)** | **N1 (%)** | **N2 (%)** | **N3 (%)** | **REM (%)** | **SL (min)** | **RL (min)** | **SE (%)** | **AI** | **PLMSI** | **AHI** | **Lowest SaO2** | **RBD** | **Sound volume (dB)** | **Frequency (Hz)** | **PPQ  (%)** | **APQ  (%)** | **Jitter  (%)** | **Shimmer  (%)** | **NHR** | **Formants** | **Harmonics** | **Wave** |
| **1** | 362.5 | 5.1 | 34.1 | 22.7 | 38.1 | 26 | 68.5 | 79.86 | 0 | 0 | 5.1 | 79 | No | 54.6 | 172.8 | 5.94 | 14.858 | 6.2 | 8.1 | 0.412 | Yes | Yes | R |
| **2** | 332.2 | 7.2 | 50.7 | 10.4 | 31.7 | 55 | 78 | 78.13 | 13 | 99 | 16.4 | 82 | Yes | 60.5 | 136.9 | 3.603 | 13.977 | 6.1 | 7.9 | 0.251 | Yes | Yes | SR |
| **3** | 228 | 9.9 | 55.7 | 30.3 | 4.2 | 1.5 | 146.5 | 71.35 | 39.2 | 0 | 49.5 | 88 | Yes | 49.5 | 183.2 | 6.912 | 18.544 | 9.4 | 12.2 | 0.363 | Yes | Yes | SR |
| **4** | 306 | 7.4 | 70.1 | 3.6 | 19 | 22.5 | 208.5 | 66.96 | 4.9 | 0 | 0 | 88 | No | 55.2 | 172.5 | 8.882 | 14.323 | 11.8 | 14.4 | 0.221 | Yes | Yes | SR |
| **5** | 260 | 10.8 | 26.5 | 34.8 | 27.9 | 11.5 | 206.5 | 55.73 | 0 | 0 | 0 | 93 | Yes | 54.5 | 200.2 | 8.262 | 25.031 | 12.9 | 15.7 | 0.388 | Yes | Yes | SR |
| **6** | 343 | 23.6 | 45.3 | 21.9 | 9.2 | 6 | 79.5 | 72.06 | 0 | 14.5 | 15.6 | 87 | Yes | 54.9 | 128.8 | 9.834 | 16.024 | 13.1 | 14.7 | 0.542 | Yes | Yes | SR |
| **7** | 208 | 31.7 | 37 | 25.2 | 6 | 32.5 | 281 | 50.21 | 8.2 | 0.3 | 29.4 | 86 | Yes | 60.3 | 266.9 | 11.224 | 25.008 | 8.8 | 13.2 | 0.539 | Yes | Yes | SR |
| **8** | 303 | 8.1 | 50.2 | 20.6 | 21.1 | 10.5 | 119.5 | 77.2 | 1.5 | 40.4 | 2.4 | 90 | Yes | 62.5 | 267.9 | 7.52 | 11.25 | 11.24 | 17.4 | 0.512 | Yes | Yes | R |
| **9** | 253.5 | 21.3 | 37.7 | 16.8 | 24.3 | 32 | 181 | 59.65 | 0.7 | 40.2 | 4 | 90 | Yes | 52.4 | 104.2 | 16.496 | 27.066 | 12.2 | 13.2 | 0.902 | Yes | Yes | R |
| **10** | 213.5 | 18.03 | 47.78 | 23.65 | 10.54 | 8 | 182.5 | 56.17 | 0 | 0 | 4.8 | 86.19 | Yes | 62.3 | 280.2 | 14.23 | 24.2 | 6.8 | 11.7 | 0.824 | Yes | Yes | SR |
| **11** | 403 | 10.5 | 49.9 | 18.2 | 21.3 | 4 | 84 | 94.4 | 13.4 | 21 | 14.1 | 83 | No | 65.9 | 220.5 | 6.82 | 28.8 | 9.87 | 16.78 | 0.412 | Yes | Yes | SR |
| **12** | 226.5 | 9.9 | 49.4 | 22.1 | 18.5 | 45.5 | 200 | 59.1 | 11.1 | 0 | 22.8 | 88 | Yes | 60.8 | 176.5 | 5.75 | 15.23 | 6.28 | 15.24 | 0.282 | Yes | Yes | SR |
| **13** | 430 | 21.9 | 58.7 | 0.1 | 19.2 | 21.5 | 87.5 | 90 | 21.9 | 0 | 102.8 | 84 | Yes | 64.4 | 180.7 | 9.56 | 17.5 | 7.85 | 23.42 | 0.114 | Yes | Yes | R |
| **14** | 290.5 | 8.6 | 67.6 | 0 | 23.8 | 9 | 144 | 69.2 | 17.8 | 152.8 | 7 | 88 | No | 59.8 | 219.2 | 7.154 | 24.885 | 11.736 | 7.154 | 0.357 | Yes | Yes | R |
| **15** | 175.5 | 34.5 | 65.5 | 0 | 0 | 25.5 | 0 | 40.5 | 24.6 | 0 | 30.8 | 85 | Yes | 66.5 | 182.2 | 6.25 | 18.24 | 5.25 | 12.52 | 0.285 | Yes | Yes | SR |
| **16** | 367 | 4 | 55.9 | 15.5 | 24.6 | 7 | 77.5 | 84.7 | 4.2 | 81.9 | 11.7 | 88 | No | 70.9 | 283.455 | 5.48 | 14.704 | 8.811 | 22.753 | 0.351 | Yes | Yes | R |
| **17** | 298 | 8.9 | 71.5 | 0 | 19.6 | 4 | 72.5 | 78.4 | 18.7 | 164 | 21.3 | 82 | Yes | 72.2 | 154.2 | 4.64 | 21.1 | 7.135 | 19.416 | 0.184 | Yes | Yes | R |
| **18** | 307 | 20.2 | 68.1 | 9.4 | 2.3 | 54.5 | 64 | 57.6 | 26.8 | 121.4 | 6.6 | 90 | Yes | 62.3 | 211.4 | 8.32 | 18.72 | 8.853 | 15.43 | 0.223 | Yes | Yes | R |
| **19** | 325 | 19.1 | 70.6 | 0 | 10.3 | 2.5 | 304 | 75.8 | 28.6 | 0 | 49.3 | 86 | No | 59.1 | 148.2 | 6.4 | 14.53 | 11.1 | 17.2 | 0.285 | Yes | Yes | SR |
| **20** | 321.5 | 70.3 | 24 | 0 | 5.8 | 20 | 213.5 | 70.7 | 40.7 | 0 | 50.2 | 83 | No | 70.0 | 140.2 | 8.2 | 21.2 | 12.2 | 16.2 | 0.201 | Yes | Yes | R |
| **21** | 312.5 | 8.3 | 67.8 | 3 | 20.9 | 9 | 73 | 88.4 | 9.6 | 0 | 10.4 | 89 | No | 75.1 | 240.3 | 10.473 | 21.2 | 9.174 | 13.4 | 0.735 | Yes | Yes | SR |
| **22** | 170 | 21.5 | 70 | 0.6 | 7.9 | 67.5 | 81 | 39 | 18.4 | 129.7 | 20.8 | 86 | Yes | 66.2 | 244.5 | 3.513 | 11.319 | 4.9 | 15.5 | 0.236 | Yes | Yes | SR |

TST, total sleep time; TIB, time in bed; N1/2/3, proportion of N1/2/3 stage; REM, proportion of REM stage; SL, sleep latency; RL, REM sleep latency; SE, sleep efficiency; AI, arousal index; PLMSI, index of periodic limb movements during sleep; AHI, apnea-hypopnea index; SaO2, saturation of oxygen; RBD, REM sleep behavior disorder; PPQ, pitch perturbation quotient; APQ, amplitude perturbation quotient; NHR, noise-to-harmonic ratio; R, rhythmic; SR, semirhythmic
